# Case Report: Combined central and peripheral nerve demyelination with neurofilament heavy chain antibodies

**DOI:** 10.3389/fimmu.2026.1734707

**Published:** 2026-02-25

**Authors:** Xue-Lu Zhao, Ze-Yu Zhao, Chun-Lin Yang, Hua Xu, Min Zhang, Guan-Qing Wang, Ying Liu, Yan-Bin Li, Rui-Sheng Duan, Xiao-Li Li

**Affiliations:** 1 Department of Neurology, Shandong Provincial Third Hospital, Cheeloo College of Medicine, Shandong University, Jinan, China; 2 Department of Neurology, The First Affiliated Hospital of Shandong First Medical University & Shandong Provincial Qianfoshan Hospital, Jinan, China; 3 Shandong Institute of Neuroimmunology, Jinan, China; 4 Shandong Provincial Key Medicine and Health Laboratory of Neuroimmunology, Jinan, China

**Keywords:** chronic inflammatory demyelinating polyradiculoneuropathy, combined central nervous system and peripheral nervous system demyelination, immunotherapy, neurofilament heavy chain antibodies, neurofilament protein

## Abstract

Combined central and peripheral demyelination (CCPD) is a rare chronic demyelinating disorder. Neurofilament (NF) proteins are structural components specific to neuronal intermediate filaments within axons. The presence of anti-neurofilament antibodies is typically associated with axonal pathology and is seldom observed in patients with CCPD. We present a case of CCPD in a patient who tested positive for immunoglobulin G (IgG) antibodies against the neurofilament heavy chain (NF-H-IgG) in both cerebrospinal fluid (CSF) and serum. Since 2019, the patient has exhibited a constellation of neurological symptoms, including severe neck and shoulder pain, left-sided limb weakness, distal extremity numbness, left-eye visual blurring, and impaired deep sensation. Electrophysiological and clinical evaluations indicated a primarily demyelinating peripheral neuropathy with secondary axonal involvement. Following treatment with low-dose corticosteroids in combination with cyclophosphamide (CTX), the patient demonstrated marked clinical improvement.

## Introduction

Combined central and peripheral nervous system demyelination (CCPD) is an inflammatory disorder characterized by the simultaneous or sequential demyelination of both the central nervous system (CNS) and peripheral nervous system (PNS). Diagnostic criteria, as defined in one study, require the following: (1) T2 hyperintense lesions in the brain, optic nerves, or spinal cord on MRI, or abnormal visual evoked potentials; (2) nerve conduction studies demonstrating conduction delays, conduction blocks, temporal dispersion, or F-wave abnormalities in at least two of the median, ulnar, or tibial nerves; and (3) the exclusion of secondary causes of demyelination (1). In most adult patients, CCPD is preceded by an infection and follows a relapsing or progressive course. It is often associated with a poor response to therapy and an unfavorable prognosis (2). A proposed pathogenesis involves a shared autoimmune response against myelin antigens or epitopes in both the central and peripheral nervous systems (3).

Neurofilament (NF) proteins are specific to neuronal axonal intermediate filaments and are essential components of the neuronal cytoskeleton (4). This family includes the neurofilament triplet proteins (light, medium, and heavy chains), α-internexin, and type III peripherin. NF proteins play critical roles in maintaining axonal caliber, stability, and elasticity; facilitating axonal transport; and promoting efficient nerve impulse conduction (5). The detection of NF-IgG in serum and cerebrospinal fluid (CSF) is indicative of axonal injury, with the presence in CSF being more specific for intrathecal synthesis (6–8). We report a case of CCPD with antibodies against the neuronal intermediate filament heavy chain (NF-H-IgG). NF-H-IgG-associated neuroimmunity may represent a novel disease spectrum.

## Case report

**Figure 1:**
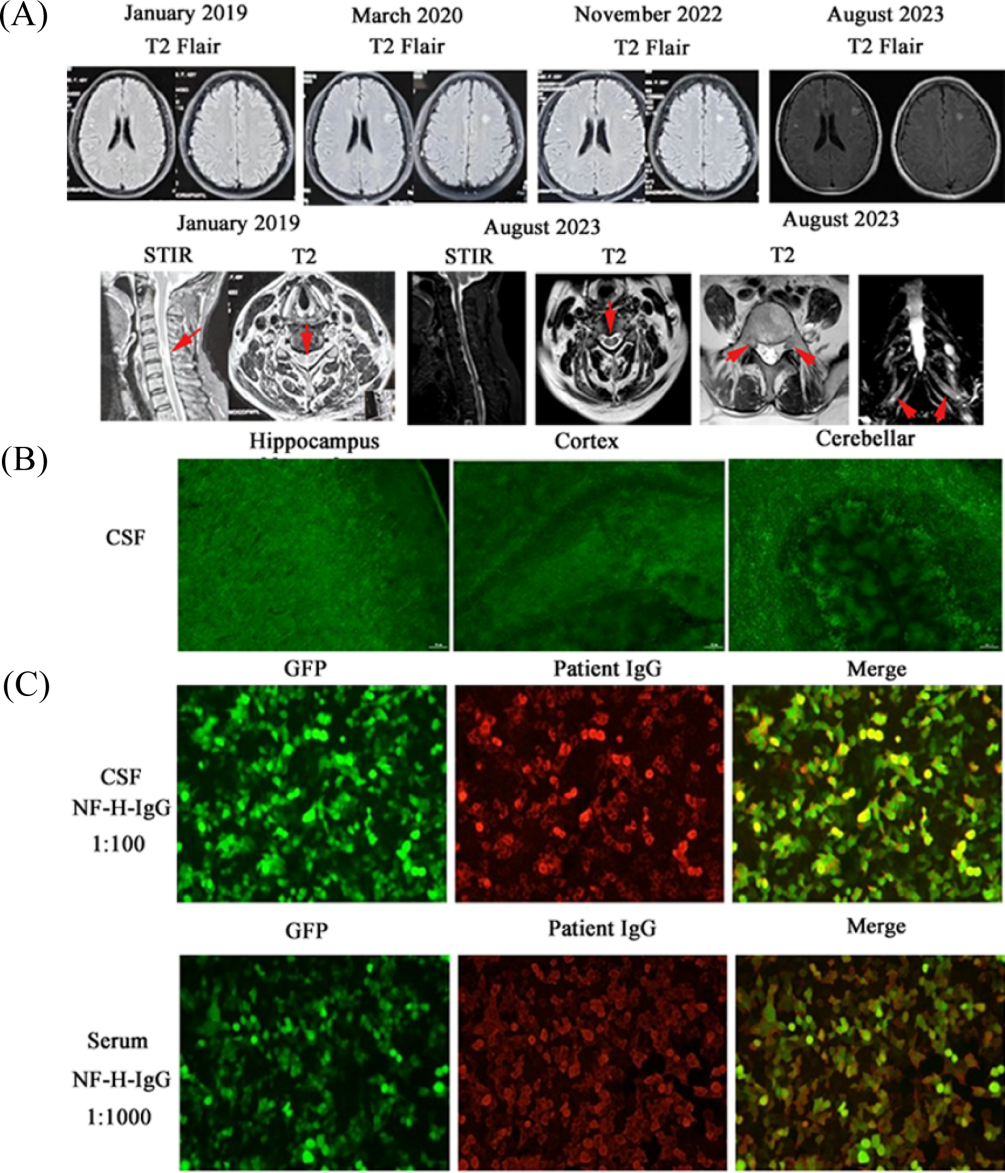
**(A)** MRI of brain, spinal cord, and lumbosacral nerve roots at different time points. Axial fluid-attenuated inversion recovery (Flair) MRI sections revealed hyperintense lesions in supratentorial white matter of both hemispheres at different time of examination. Sagittal short tau inversion recovery (STIR) and coronal T2-weighted images of the cervical spine showed focal T2-hyperintense cord lesion in Janury 2019 and August 2023 (C4-6). Coronal T2-weighted image and the reconstruction of lumbosacral nerve roots showed multiple thickened lumbar nerve roots. **(B)** Serum and CSF antibodies were verified positive in rat hippocampus, cortex, and cerebellar using tissue-based assays (TBA). Especially, neurofilamentous fluorescence surrounds Purkinje cells and extends into molecular layer in cerebellar, suggesting the possibility of anti-NF antibodies. **(C)** NF-H-IgG were assessed by cell-based assay (CBA) with immunofluorescence double staining. NF-H-IgG positive in both CSF (1:100) and serum (1:1000). GFP, green fluorescent protein.

In January 2019, a 55-year-old woman presented with severe posterior neck and shoulder pain. Cranial and Cervical Spinal Cord Magnetic Resonance Imaging (MRI) revealed multiple patchy lesions in bilateral frontal lobes. These lesions exhibited slight hyperintensity on T2-weighted imaging (T2WI), slight hypointensity on T1-weighted imaging (T1WI), and high signal intensity on T2-weighted Fluid-Attenuated Inversion Recovery (T2-FLAIR), diffusion-weighted imaging (DWI), and apparent diffusion coefficient (ADC) sequences, with no significant enhancement observed on post-contrast scans. Additionally, at the C4–C6 vertebral levels, a T2WI hyperintense signal was noted in the central-posterior portion of the spinal cord. (**Figure 1A**). Serum testing was negative for aquaporin-4 (AQP4) IgG and myelin oligodendrocyte glycoprotein (MOG) IgG. No oligoclonal bands (OBs) were detected in either serum or cerebrospinal fluid (CSF) (**Table 1**). She was treated with pulse steroid therapy followed by a tapering regimen, which led to complete resolution of the neck and shoulder pain.

**Table 1:** Clinical characteristics and laboratory results.

Time contents		January 2019	March 2020	November 2022	August 2023
Clinical symptoms		Severe pain in neck and both shoulders	Left limb weakness, numbness in hands and feet	Blurred vision in left eye and numbness in extremities	Severe numbness in extremities
INCAT		NA	4	2	2
EDSS		NA	5.5	3.5	4
Serum	AQP4-IgG	Neg(ELISA)	Neg(CBA)	Neg(CBA)	NA
	MOG-IgG	Neg(IIFT)	Neg(CBA)	Neg(CBA)	NA
	GFAP-IgG	NA	Neg(CBA)	Neg(CBA)	Neg(CBA)
	OBs	Neg	Neg	Neg	NA
	M protein	NA	NA	NA	Neg
	NF155/NF186/CNTN1/ CASPR1/MAG/CNTN2-IgG	NA	NA	NA	Neg(CBA)
	αIN-IgG	NA	NA	NA	Neg(CBA)
	NF-H-IgG	NA	NA	NA	1:1000(CBA)
	NF-L-IgG	NA	NA	NA	Neg(CBA)
CSF	Cell count (*10^6^/L)	NA	10	Normal	0
	Protein (mg/mL)	NA	80	72.1	69.9
	GFAP-IgG	NA	NA	NA	Neg(CBA)
	OBs	Neg	Neg	Neg	NA
	αIN-IgG	NA	NA	NA	Neg(CBA)
	NF-H-IgG	NA	NA	NA	1:100(CBA)
	NF-L-IgG	NA	NA	NA	Neg(CBA)
Treatment		GCs (from 500 mg to 0)	GCs (from 500 mg to 0)	GCs	GCs
				(from 500 mg to 16 mg)	
				MMF	CTX

INCAT, Inflammatory Neuropathy Cause and Treatment; NA, Not available; EDSS, Expanded Disability Status Scale; AQP4, Aquaporin 4; IgG, Immunoglobulin G; Neg, Negative; ELISA, Enzyme-Linked Immunosorbent Assay; CBA, Cell-Based Assay; MOG, Myelin oligodendrocyte glycoprotein; IIFT, Indirect Immunofluorescence Technique; GFAP, Glial ﬁbrillary acidic protein; NF155, Neurofascin 155; NF186, Neurofascin 186; CNTN1, Contactin-1; CASPR1, Contactin-associated protein 1; MAG, Myelin-associated glycoprotein; CNTN2, Contactin-2; αIN, alpha internexin; NF-H, Neuroﬁlament heavy chain; NF-L, Neuroﬁlament light chain; CSF, Cerebrospinal ﬂuid; OBs, Oligoclonal bands; GCs, Glucocorticoids; MMF, Mycophenolate mofetil; CTX, Cyclophosphamide.

In March 2020, the patient experienced left limb paralysis and numbness in the hands and feet. Neurological examination revealed the following positive signs: grade 4 muscle strength in the left limbs, impaired deep sensation, and a positive Romberg’s sign, with an Inflammatory Neuropathy Cause and Treatment (INCAT) disability score of 4 (3 in the upper limbs and 1 in the lower limbs) and an Expanded Disability Status Scale (EDSS) score of 5.5 (ambulation: 4, visual: 0, brainstem: 0, pyramidal: 3, cerebellar: 2, sensory: 2, bowel/bladder: 0, cerebral: 0). CSF analysis demonstrated a leukocyte count of 10 × 10^6^/L and an elevated protein level of 80 mg/dL. Repeat serological tests remained negative for AQP4-IgG, glial fibrillary acidic protein (GFAP)-IgG, and MOG-IgG. OBs were again negative in both serum and CSF (**Table 1**). Cranial MRI revealed multiple punctate lesions exhibiting long T1WI and long T2WI signals in the subcortical areas of the bilateral frontal and parietal lobes, the right medulla oblongata, and the corona radiata. These lesions appeared hyperintense on T2-FLAIR. The lesion in the left frontal lobe showed slightly increased signal on DWI and high signal on the ADC map. No obvious enhancement was noted on post-contrast scans. Electromyography (EMG) indicated involvement of multiple motor and sensory nerves in the extremities, consistent with a mixed pattern of demyelination and axonal injury; somatosensory evoked potentials (SEP) in the lower limbs showed poorly defined waveforms. The patient received another course of pulse steroid therapy with subsequent tapering, and ambulation function improved significantly, with the corresponding EDSS score decreasing from 4 to 2. Given the patient’s prompt and marked response to pulse steroid therapy during this episode, coupled with negative results for serum AQP4-IgG, GFAP-IgG, and MOG-IgG upon re-testing, and OBs again negative in both serum and CSF, long-term immunosuppressive therapy was not initiated at other hospital.

In November 2022, the patient experienced a recurrence, presenting with blurred vision in the left eye and numbness in the extremities. Visual evoked potential (VEP) testing showed prolonged P100 latency bilaterally, more pronounced on the left side. Visual acuity was 1.0 in the right eye and 0.8 in the left eye. Other ophthalmological assessments, including optical coherence tomography (OCT), were within normal limits. The patient had an INCAT score of 2 (1 point for the upper limbs and 1 point for the lower limbs) and an EDSS score of 3.5 (ambulation: 2, visual: 1, brainstem: 0, pyramidal: 1, cerebellar: 2, sensory: 2, bowel/bladder: 0, cerebral: 0). Brain MRI was unchanged from prior studies (**Figure 1A**). CSF analysis at this time revealed a normal cell count but an elevated protein level of 72.1 mg/dL. Serological tests remained negative for AQP4-IgG, GFAP-IgG, and MOG-IgG, and no oligoclonal bands were detected in either serum or CSF (**Table 1**). In consideration of the relapsing-remitting disease course and the patient’s financial situation, mycophenolate mofetil (MMF) was initiated as long-term immunosuppressive maintenance therapy, with the goals of minimizing corticosteroid-related adverse effects and preventing disease recurrence. This treatment resulted in an alleviation of blurred vision, though without a significant change in the EDSS score.

In August 2023, the patient reported worsening numbness in the extremities. Physical examination revealed weakened tendon reflexes, diminished pain sensation in the limbs, and a positive Romberg’s sign. The INCAT score remained stable compared to the previous assessment, while the EDSS score was assessed at 4 (ambulation: 2, visual: 1, brainstem: 0, pyramidal: 1, cerebellar: 2, sensory: 3, bowel/bladder: 0, cerebral: 0). Cranial and cervical spinal cord MRI findings were unchanged from the previous study. (**Figure 1A**). Nerve conduction studies (NCS) showed reduced motor nerve conduction velocities in multiple nerves (**Table 2**). Temporal dispersion was observed, along with a probable conduction block between the axilla and Erb’s point of the left median nerve. Sensory nerve action potential (SNAP) amplitudes were reduced or absent, though sensory conduction velocities remained normal (**Table 2**). F-wave latencies in the upper and lower limbs were markedly prolonged with reduced persistence (**Table 2**). Needle electromyography (EMG) revealed acute denervation potentials (positive sharp waves and fibrillation potentials) and chronic reinnervation patterns (complex repetitive discharges and long-duration, high-amplitude, polyphasic motor unit action potentials) in the bilateral tibialis anterior and first interosseous muscles. The skin sympathetic response (SSR) was abnormal, indicating autonomic involvement, while somatosensory evoked potentials failed to elicit reproducible waveforms. Serum biochemical and tumor markers were within normal limits except for a mildly elevated carcinoembryonic antigen level (6.23 ng/mL). Serum M-protein was negative, and blood glucose levels were normal. The patient denied any family history of neurological disease. Repeat CSF analysis showed acellular fluid with an elevated protein level of 69.9 mg/dL (**Table 1**). Multiple nodular T2WI hyperintensities were observed in the bilateral lumbosacral plexus, with no significant enhancement on post-contrast imaging. (**Figure 1A**). Nerve ultrasound demonstrated uneven, multifocal, and thickened nerves—including the median, ulnar, radial, sciatic, and tibial nerves—with an ultrasound pattern sum score > 10, supporting the diagnosis of chronic inflammatory demyelinating polyradiculoneuropathy (CIDP). Tissue-based assay (TBA) detected autoantibodies in the CSF that bound to the rat hippocampus, cortex, and cerebellum (**Figure 1B**). Notably, neurofilamentous fluorescence was observed surrounding Purkinje cells and extending into the molecular layer of the cerebellum, suggesting the presence of anti-neurofilament antibodies. Subsequent cell-based assay (CBA) confirmed NF-H-IgG in both CSF (titer 1:100) and serum (titer 1:1000) (**Figure 1C**). Both serum and CSF were negative for anti-GFAP, AP3B2, ARHGAP26, ATP1A3, CARP VIII, CASPR2, GAD65, GluK2, GluRδ2, Homer3, IgLON5, ITPR1, KLHL11, mGluR1, mGluR2, αIN, NF-L, PCA2, PKCγ, Septin-5, TRIM9, TRIM67, NF155, NF186, CNTN1, CASPR1, MAG, and CNTN2 auto-antibodies (tested by CBA). Following a final diagnosis of CCPD with positive NF-H-IgG, the patient was maintained on a regimen of low-dose corticosteroids and cyclophosphamide (CTX).

**Table 2:** Characteristics of nerve conduction studies.

Test results	Normal	Aug 2023	Rate* (%)	Jan 2024	Rate* (%)
Median nerve (L/R)					
DML (ms)	≤4.1	3.3/4.1		3.3/3.8	
MCV (m/s)	≥49.0	38.7/38.6	21.0/21.2	41.0/35.5	16.3/27.6
CMAP amplitude (mV)	≥7.0	7.3/7.7		7.1/7.3	
F wave latency (ms)	≤30.0	36.2/36.5	20.7/21.7	35.5/36.8	18.3/22.7
F-wave persistence (%)	≥74.0	20.0/75.0		100.0/100.0	
SCV (m/s)	≥41.8	48.3/49.8		Unobtainable	
SNAP amplitude (uV)	≥12.7	6.4/5.7		NR	
Ulnar nerve (L/R)					
DML (ms)	≤3.2	2.8/2.5		2.7/2.8	
MCV (m/s)	≥61.0	48.3/48.4	20.8/20.7	32.4/48.7	46.9/20.2
CMAP amplitude (mV)	≥6.0	10.0/5.4		11.6/8.4	
F wave latency (ms)	≤30.0	40.1 (L)	33.7(L)	37.9/41.8	26.3/39.3
F-wave persistence (%)	≥74.0	45.0 (L)		100.0/100.0	
SCV (m/s)	≥53.7	53.2/52.1		Unobtainable	
SNAP amplitude (uV)	≥4.9	4.1/4.0		NR	
Tibial nerve (L/R)					
DML (ms)	≤5.1	5.9/4.1	15.7 (L)	3.6/3.7	
CMAP amplitude (mV)	≥4.0	1.6/2.8		2.1/1.9	
F wave latency (ms)	≤50.0	74.8 (R)	49.6 (R)	64.1/61.9	28.2/23.8
F-wave persistence (%)	100.0	60.0 (R)		100.0/100.0	
Common peroneal nerve (L/R)					
DML(ms)	≤3.6	5.9/8.3	63.9/130.5	4.6/7.0	27.7/94.4
MCV (m/s)	≥41.6	35.5 (L)	14.7 (L)	36.5/27.8	12.3/33.2
CMAP amplitude (mV)	≥3.0	1.6/0.7		3.5/0.6	

*Rate: the percentage deviation (reduction or elevation) of the patient's results from normal ranges. Values fulfilling the 2021 EAN/PNS diagnostic criteria for CIDP are marked in red. .

L, left; R, right; DML, distal motor latency; MCV, motor conduction velocity; CMAP, Compound muscle action potentials; SCV, sensory conduction Velocity; SNAP, sensory nerve action potentials; NR, no response.

At a follow-up evaluation during a CTX cycle in January 2024, examination demonstrated muscle strength of 4+/5 in all four limbs, deep sensory loss, and a positive Romberg’s sign. Tendon reflexes were traceable in the upper limbs but absent in the lower limbs. The INCAT score remained unchanged. The EDSS score was 3 (ambulation: 1, visual: 0, brainstem: 0, pyramidal: 2, cerebellar: 2, sensory: 2, bowel/bladder: 0, cerebral: 0). Imaging and electrophysiological studies (cranial/cervical MRI, EMG) were substantially unchanged from prior assessments. SNAPs could not be elicited in the bilateral upper limbs. The skin sympathetic response (SSR) was now normal in the extremities; however, SEP in the lower limbs exhibited clear waveform morphology but a significantly delayed P38 latency, aligning with the clinical deep sensory deficit. Following 20 months of follow-up, marked improvement in the numbness was observed.

## Discussion

This case was characterized by intracranial demyelination, spinal cord lesions, and peripheral neuropathy. Electrophysiological studies revealed reduced motor nerve conduction velocities, waveform dispersion, and conduction block—features consistent with acquired demyelination. Plasma cell proliferation-associated peripheral neuropathy and autoimmune Ranvier’s node disease were both excluded. The electrophysiological and clinical profile of the peripheral neuropathy supported a diagnosis of classic chronic inflammatory demyelinating polyneuropathy (CIDP). Subsequently, a diagnosis of CCPD was established.

CCPD is defined by concurrent demyelination in the central and peripheral nervous systems and is occasionally associated with neurofascin 155-IgG (NF155-IgG) (1, 9). In this patient, NF-H-IgG was detected in both CSF and serum. NF antigens may become exposed in contexts such as malignancy, systemic infection, or immune checkpoint inhibitor therapy (6). The presence of NF-IgG in serum or CSF suggests axonal dysfunction, with intrathecal positivity being more specific (6–8). Reportedly, CSF-defined NF-IgG autoimmunity often presents with encephalopathy, cerebellar ataxia, and myeloradiculoneuropathies—conditions in which NF-L, NF-H, and α-internexin are highly expressed—and typically responds to immunotherapy (6–8). While NF-L-IgG has been linked to length-dependent axonal neuropathy in some reports (10), the current case exhibited predominantly demyelinating neuropathy with secondary axonal damage. It remains uncertain whether NF-H-IgG in this patient is directly related to secondary axonal injury or represents an epiphenomenon. Notably, NF-L-IgG has been associated with small cell lung cancer and Merkel cell carcinoma, among other neuroendocrine tumors, whereas NF-H-IgG may be linked to lymphomas (6, 7). In consideration of her clinical and socioeconomic context, the patient was treated with low-dose glucocorticoids and cyclophosphamide. After 20 months, she showed significant improvement in limb numbness.

It is worth noting that neurofilaments are intracellular proteins, and the mechanism by which NF-IgG antibodies access their targets across the cell membrane remains unclear. NF-IgG may serve as a non-pathogenic biomarker reflecting T cell-mediated axonal injury rather than directly causing disease (6). This case expands the clinical spectrum of CCPD associated with NF-H-IgG and underscores the need for further investigation into its underlying mechanisms.

This retrospective analysis has several limitations. Firstly, detailed neurological examination records and the initial CSF cell count results from the patient’s early presentation at a local hospital are unavailable. Secondly, the primary early symptom—neck and shoulder pain—was not assessed using standardized pain scales. Additionally, orbital MRI was not performed when vision-related symptoms emerged during the disease course. These factors may have affected the comprehensive documentation and precise evaluation of early-stage changes. Nevertheless, the long-term, systematic follow-up data of this case and its association with specific antibodies (NF-H-IgG) still provide valuable insights into the clinical and immunological characteristics of this rare disease.

## Data Availability

The raw data supporting the conclusions of this article will be made available by the authors, without undue reservation.
